# Acoustic black hole effect enhanced micro-manipulator

**DOI:** 10.1038/s41378-024-00789-z

**Published:** 2024-10-12

**Authors:** Qiu Yin, Haoyong Song, Zhaolong Wang, Zhichao Ma, Wenming Zhang

**Affiliations:** 1 State Key Laboratory of Mechanical System and Vibration, Shanghai Jiao Tong University, Shanghai, China; 2 Institute of Medical Robotics, School of Biomedical Engineering, Shanghai Jiao Tong University, Shanghai, China; 3 College of Mechanical and Vehicle Engineering, Hunan University, Changsha, China; 4 School of Energy Science and Engineering, Harbin Institute of Technology, Harbin, P. R. China

**Keywords:** Electrical and electronic engineering, Nanofluidics

## Abstract

Microparticle manipulation is a critical concern across various fields including microfabrication, flexible electronics and tissue engineering. Acoustic-activated sharp structures have been designed as simple and flexible tools to manipulate microparticles with their good compatibility, fast response, and broad tunability. However, there still lacks rational acoustic-structure design for effective energy concentration at the acoustic-activated sharp structures for microparticle manipulation. Here, we present the acoustic black hole (ABH) effect as enhancement for the acoustic micro-manipulator. It provides great reliability, simplicity and ease of use, supporting custom design of high-throughput patterning modes. Moreover, compared to commonly used configurations, such as cylindrical or conical microneedles, those microneedles with ABH profile exhibit superior acoustic energy focusing at the tip and induce stronger acoustofluidic effects. The average acoustic flow velocity induced by the ABH microneedle is 154 times greater than that of the conical one and 45 times greater than that of the cylindrical microneedle. Besides, the average acoustic radiation force (ARF) produced by the ABH microneedle against acrylic microparticles is about 319 times greater than that of the cylindrical one and 16 times greater than that of the conical one. These results indicate that ABH design significantly enhances microparticle manipulation. We demonstrate this concept with ABH effect enhanced microparticle manipulation and study the parameters influencing its performance including operating frequency, operating voltage and particle diameter. Furthermore, considering the flexibility of this system, we employ it for various patterning and high-throughput microparticle manipulation. This work paves the way for controllable microparticle manipulation, holding great potential for applications in microfabrication and biomedicine.

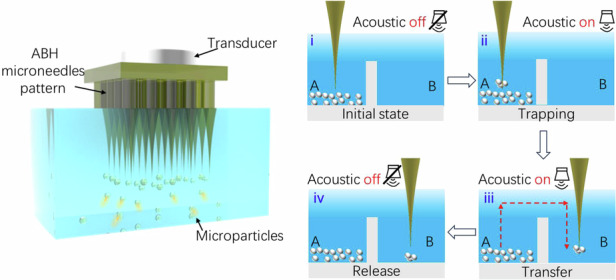

## Introduction

Manipulation of microparticles (e.g. metal nanowires^1,2^, carbon nanotubes^3,4^, colloidal particles^5,6^, cells^7,8^ and cellular spheroid^9,10^) is crucial for both fundamental research and industry, including microfabrication^11,12^, flexible electronics^13^ and tissue engineering^14,15^. With the rapid development of micro- and nano-systems, numerous microparticle manipulation technologies have emerged^16–18^. For instance, Ashkin et al.^19^ invented the Nobel prize-winning technology of optical tweezers, demonstrating that microparticles can be trapped and manipulated using optical beams. Additionally, there are methods employing other external physical fields, including electrical^20,21^, magnetic^10,22,23^, and acoustic field^24,25^. Among these, acoustic methods show advantages of good compatibility, fast response, contact-free and broad tunability^26–28^.

In pursuit of simple and flexible manners with high selectivity, the acoustic activated sharp structures have been widely used for particle manipulation^29–33^. These structures are designed to focus acoustic energy at the sharp tip, enabling the capture and movement of particles. For example, Hsu et al.^34^ and Fei et al.^35^ developed the needle-shape acoustic transducers, where the acoustic energy emitted from the sharp tips traps and moves the microspheres by acoustic radiation force. These studies show the high selectivity and flexibility of needle-shape acoustic manipulators. However, they require the costly manufacturing of miniaturized and high-frequency transducers. A recent study by Hu et al.^36,37^ demonstrated an acoustic manipulator made of stainless steel needle driven by sandwich type piezoelectric transducer, capable of transporting small particles. Moreover, in a subsequent work, they added a glass fiber with a diameter of 10 μm to the end of needle system^38^. enabling the manipulation of particles down to micrometer scale. These low-cost acoustic manipulators demonstrate the feasibility of micro-manipulation based on a vibrating sharp structure, however, still require further optimization in acoustic-structure design. For instance, the robustness of manipulation depends on the acoustic streaming generated from the vibrating structures, meaning that poorly designed structures result in a frequency-dependent and unstable manipulation process. Thus, there is an urgent requirement for reliable and easy-to-use acoustic manipulator, necessitating the optimized design of acoustic energy harvesting at the sharp structure.

Acoustic black hole (ABH), an innovative acoustic-structure design conception, shows promise in this regard. It relies on profile design with a spatial power-law distribution in thin-walled structures (usually cantilever or plates). This design has been widely applied in the fields of vibration and acoustics^39,40^, including vibration mitigation^41,42^, phononic crystals^43,44^, and energy harvesting^45,46^. The propagation of elastic waves can be controlled and focused at specific locations by designing the shape of the structure, thereby enhancing the local vibration. Recent study on the use of ABH structures for manipulating macroscopic particles such as shrimp eggs and grass seeds have shown the potential application of ABH structures in acoustic tweezers^32^. However, to our knowledge, no research currently investigates the acoustofluidic effect of ABH-designed micro-sized needle tips on microparticles suspended in fluid. In particular, how to take advantage of relevant properties to enhance the acoustic-structure design for microparticle manipulation.

In this article, we design and demonstrate an ABH microneedle acoustofluidic system for microparticle manipulation, leveraging the design conception of ABH to focus acoustic energy at the tip. The mechanism of this system was systematically investigated through both simulations and experiments. The numerical simulation results reveal that the ABH-designed structure enhances acoustic energy at the microneedle tip, providing higher manipulation efficiency comparison to conical or cylinder microneedles. Furthermore, we experimentally verified this phenomenon. The results show that our system can trap and transfer the microparticles in a simple manner. Moreover, considering the versatility of 3D printing technology, we customized and produced these microneedles into an array-integrated system with specific shapes as needed. Furthermore, we employed the system to achieve high-throughput patterning of microparticles. Therefore, this study paves the way for further exploration of ABH in microparticle manipulation. The excellent performance of the present system demonstrates the great potential for use in various applications, such as bioassembly^47^, additive manufacturing^48^, and micro robots^49,50^.

## Conception of ABH microneedle acoustofluidic system

**Fig. 1:**
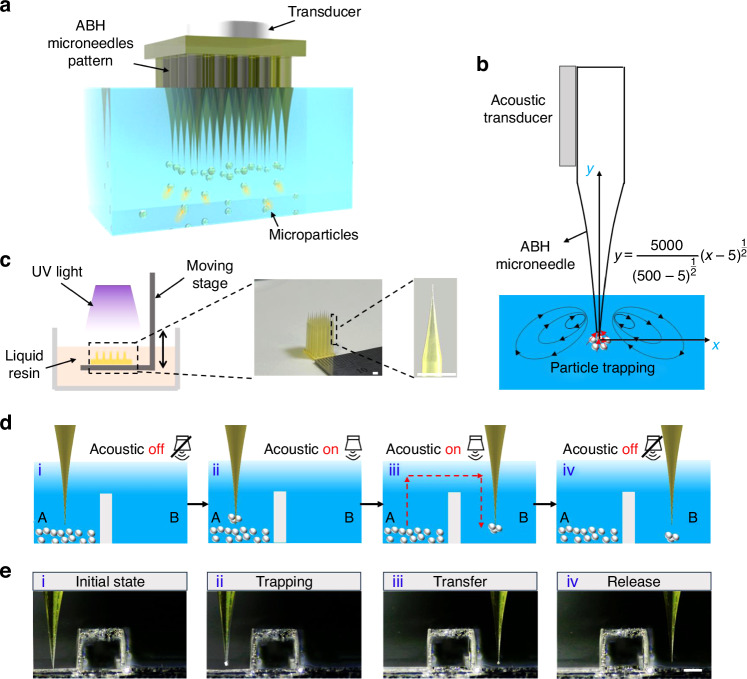
The ABH microneedle acoustofluidic system for microparticle manipulation. **a** The schematic of ABH microneedle acoustofluidic system for high-throughput patterning of microparticles. **b** The schematic of the ABH microneedle acoustofluidic system operating principle. **c** The schematic and results of the 3D printing process for ABH microneedle fabrication. Scale bar: 2 mm. **d** The schematic of microparticle manipulation process of ABH microneedle acoustofluidic system. **e** The microparticle manipulation process of ABH microneedle acoustofluidic system. Scale bar: 1 mm

As shown in Fig. 1a, we designed and presented an ABH microneedle acoustofluidic system for high-throughput microparticle manipulation with patterned distributions. The operation principle of this ABH microneedles acoustofluidic system is illustrated in Fig. 1b. The system consists of three simplex components: an ABH microneedle, a PZT transducer and a liquid container. When the PZT transducer is driven by a signal generator and voltage amplifier, it generates acoustic waves that propagate through the microneedle to its tip. As the microneedle is designed with a power-law decreasing thickness profile, the velocity of the incoming elastic wave is gradually reduced close to zero as the size from the root to the tip is decreased. Consequently, acoustic energy is focused at the tip with near-zero reflection^39^. Furthermore, when the acoustic energy transferred into the liquid phase, it induces strong acoustic streaming and ARF on the microparticle. This results in the trapping of microparticles at the ABH microneedle’s tip.

In order to fabricate the ABH microneedle, we employed the projection micro-stereolithography (PμSL) 3D printing technique. The schematic of the 3D printing is shown in Fig. 1c. The 3D printing process are based on three steps. Firstly, the 3D model was established using a computer-aided design (CAD) software. Secondly, the 3D model was sliced into several 2D images using slicing software. Finally, 2D images were transformed into 405 nm UV light patterns, and the solidification of the material in the exposed area occurred to form each layer. The projection of each image was followed by lowering a moving stage. This process proceeded repeatability until the entire structure was fabricated on the platform. Finally, we can fabricate the ABH microneedles with the diameter in the tip of 10 μm, a top diameter of 1000 μm, and a height of 5000 μm.

Afterwards, we demonstrated the use of this ABH enhanced acoustofluidic system for trapping and manipulation microparticles to desired location as shown in Fig. 1d, e. Firstly, the ABH microneedle was inserted into the solution containing PMMA microparticles. Then, we moved the ABH microneedle close to the microparticles and turned on the acoustic transducer, which could trap the microparticles at the tip. Subsequently, we transferred this acoustic activated ABH microneedle in the desired path. Finally, we turned off the acoustic transducer to release the microparticles. This process successfully demonstrates the capability of the system for precise microparticle transfer.

## Results and discussions

**Fig. 2:**
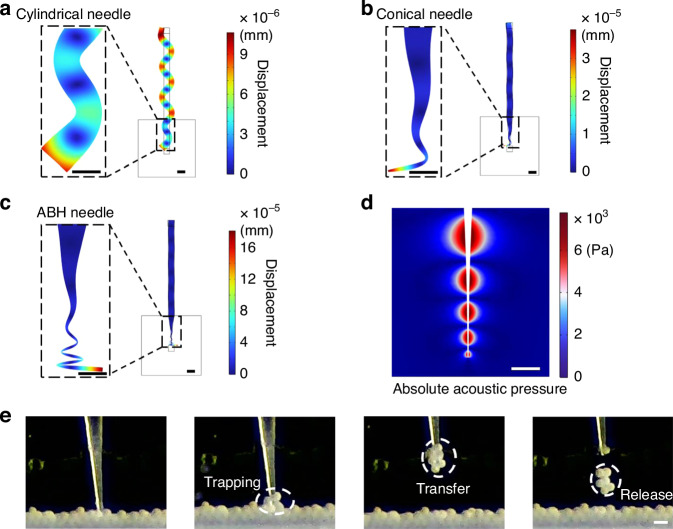
The vibration characteristics of the microneedles. **a** Numerical simulation shows the distribution of vibration displacements along cylindrical microneedle. Scale bar: 1 mm. **b**, **c** Numerical simulation shows that the vibration amplitude gradually becomes larger along the conical/ABH microneedle. Scale bar: 1 mm. **d** Numerical simulation shows the acoustic pressure in the liquid of ABH microneedle. Scale bar: 50 μm. **e** ABH microneedle shows good performance in microparticle trapping and transfer. Scale bar: 100 μm

We conducted numerical simulations and experiments to investigate the mechanism and performance of the ABH microneedle. Firstly, the vibration characteristics of the cylindrical microneedle, conical microneedle and ABH microneedle were studied. The numerical simulation results illustrated in Fig. 2a–c, reveal differences in the vibration modes among these three microneedles. Figure 2a shows cylindrical microneedle exhibits multiple areas with uniform vibration amplitude along its length, with maximum values being consistent. In contrast, the vibration amplitude gradually increased as it transmitted from the root to the tip for both conical microneedle and ABH microneedle. Notably, compared with conical microneedles and cylindrical microneedles, the ABH structure exhibits the most obvious enhancement effect at the microneedle tip. Further, we studied the absolute acoustic pressure field in the water domain for ABH needle. As depicted in the Fig. 2d of acoustic pressure distribution in the water domain, the local strongest acoustic pressure region corresponds to the maximum vibration region shown in Fig. 2c. Taking advantage of this feature, we then used the ABH microneedle to capture microparticles. As shown in Fig. 2e, the microparticles were successfully trapped at the tip of the ABH microneedle when the acoustic was activated and could be transferred to the desired position. When the acoustic was turned off, the microparticles can be released from the microneedle. However, the cylindrical microneedle only induced weak acoustic streaming to disrupt the microparticles but not trap them as shown in Fig. S1.

**Fig. 3:**
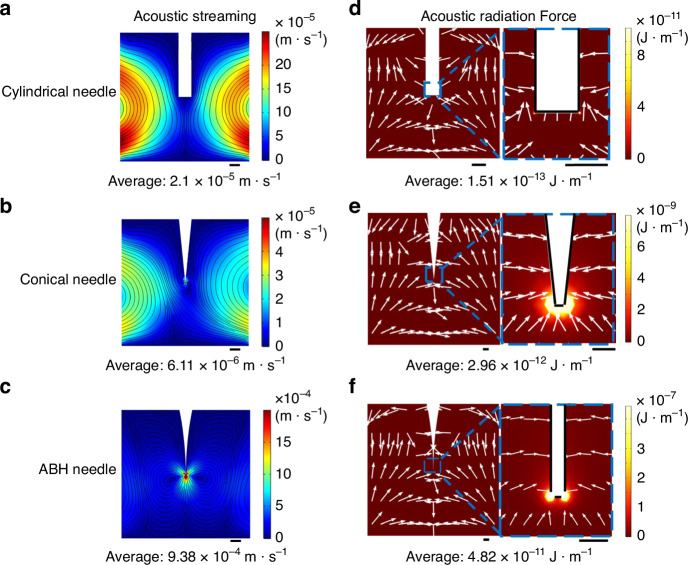
The acoustofluidic effects of the microneedles. **a**–**c** Numerical simulation shows the acoustic streaming in liquid for cylindrical microneedle, conical microneedle, ABH microneedle, respectively. **d**–**f** Numerical simulation shows the acoustic radiation force act on the acrylic microparticles in liquid for cylindrical microneedle, conical microneedle, ABH microneedle, respectively. Scale bars are 1 mm in (**a**), (**d**), 50 μm in (**b**), (**c**), 20 μm in (**e**) and (**f**)

Besides investigating the vibration characteristics of microneedles, we also studied two primary acoustofluidic effects: acoustic radiation force and acoustic streaming. We conducted numerical simulations to analyze the behavior of an acoustic activated ABH microneedle within water medium, aiming to elucidate the mechanisms underlying microparticle trapping. The acoustic streaming actively pushed the microparticle around the microneedle tip by the drag force. And the microparticles dispersed in the fluid were subject to ARF that converged toward the microneedle tip. Moreover, under the same conditions, the average acoustic flow velocity induced by the ABH needle was 154 times than that of the conical needle and 45 times than that of the cylindrical needle as shown in Fig. 3a–c. The results also show that the average ARF produced by the ABH needle against particles was about 319 times than that of the cylindrical needle and 16 times than that of the conical needle as shown in Fig. 3d–f. These results indicate that the ABH enhances the performance of acoustic microparticle manipulator.

**Fig. 4:**
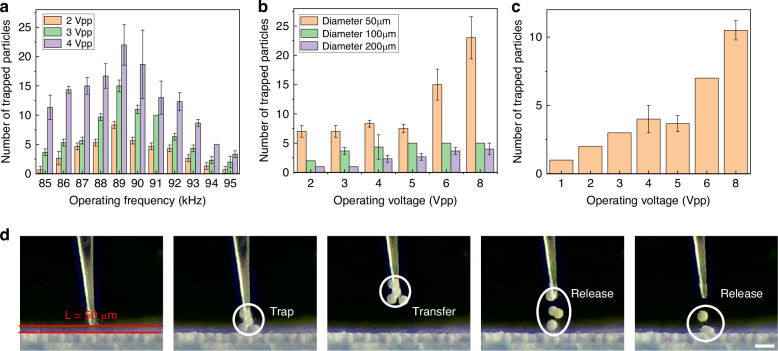
The factors influencing the effect of acoustofluidic manipulation. **a**, **b** The influence of operating frequency, voltage and particle diameter on the number of trapped particles. **c** The influence of operating voltage when the distance from microneedle tip to microparticle is 50 μm. **d** The noncontact trapping property of the ABH microneedle. Scale bar: 100 μm

As the acoustofluidic manipulation relies on both acoustic radiation force and acoustic streaming, we investigated the factors influencing these effects, including operating frequency, operating voltage and particle diameter. As shown in Fig. 4a, we can find that when the operating frequency from 85 kHz to 95 kHz, the number of trapped microparticles first increases and then decreases, and reach the maximum around 89 kHz, which is close to the resonance frequency as shown in Fig. S2. Therefore, we selected the operating frequency of 89 kHz for subsequent manipulation experiments. Afterwards, we discussed the effects of operating voltage and particle size on the number of captures as shown in Fig. 4b. The results show that the number of captured microparticles increases with increasing the operating voltage, which is due to the increase in the acoustic radiation force acting on the particles. ABH microneedles can capture microparticles of different sizes, and the number of small diameter particles is much greater than that of large diameter particles. Besides, ABH microneedles can also capture microparticles remotely as shown in Fig. 4d. When the microneedle is 50 μm away from the particles, the particles can also be attracted and captured. And the number of captured microparticles is also positive correlated to the working voltage as shown in Fig. 4c.

**Fig. 5:**
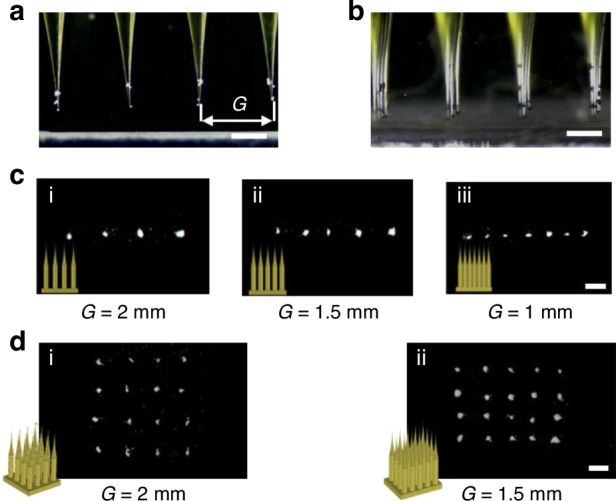
Performance of the integrated ABH microneedles. The integrated ABH microneedles used for high-throughput and controllable microparticle patterning such as (**a**) and (**c**) line mode and (**b**) and (**d**) plane mode with different gap. Scale bar: 1 mm

Leveraging the versatility of 3D printing technology, we developed microneedles designed for precise manipulation of microparticles based on user requirements. We assessed the ability to efficiently transfer microparticles using microneedle array for high-throughput, controlled patterning. We demonstrated two primary modes of microparticle manipulation in our experiments. Firstly, as shown in Fig. 5a, the microparticle can be patterned in lines with designed distances. And the gap between two microneedles can be tuned from 1 mm to 2 mm as shown in the Fig. 5c. Besides, microparticle can be manipulated in array as shown in Fig. 5b, we achieve dexterous control of microparticle arrangement and patterning through controlling the gap between two microneedles and the shape of the plane as shown in Fig. 5d.

## Conclusions

In summary, we have performed a microparticle manipulator based on the acoustic activated microneedle with ABH profile, which exhibits notable reliability, simplicity, and ease of use in microparticle manipulation. This system also allows for custom-designed high-throughput patterned modes. First of all, we investigated the mechanisms by which ABH microneedles enhance microparticle manipulation. Numerical simulations revealed that microneedles with ABH profile exhibit superior acoustic energy focusing capabilities at the tip and induce stronger acoustofluidic effects in comparison with cylindrical needles and conical needles. This finding was subsequently verified experimentally, and we explored factors affecting manipulation, including particle size, voltage amplitude, frequency, and the distance between the particle and the microneedle tip. Furthermore, since the microparticle patterns are determined by the device configuration, which can be easily altered by changing the microneedle arrangements, the system is well-suited for high-throughput microparticle manipulation. Overall, ABH enhanced manipulator is an innovative, straightforward, and efficient setup for microparticle manipulation. Its superior performance in trapping, transferring, and patterning microparticles highlights its promising potential across various fields, including biofabrication, flexible electronics, and disease diagnosis. Future studies are expected to consider the liquid viscosity, whose variations are common among different biofabrication media. To ABH effect, the liquid act as an energy dissipator, which can influence the vibration amplitude^51–55^.

## Material and methods

### Device fabrication

To fabricate the ABH microneedle acoustofluidic system, we designed and fabricated the ABH microneedles by the PμSL 3D printing technique (P140, BMF Material Technology Inc., China) with the x-y feature resolution of 7.56 μm and layer height of 10 μm at first. Then, the ABH microneedle was glued to the center of the PZT transducer (Pz4, COSSON) with the tip extending about 1.5 cm beyond the transducer. Besides, in order to precisely control the position of the micro manipulator, it is fixed on a three-dimensional mobile platform. During the working process, the PZT transducer was driven by a signal generator (DG1022Z, RIGOL) connected to a voltage amplifier (WMA-300, Falco System). To characterize the microparticle manipulation and pattern process of the aoustofluidic system, we selected the polymethylmethacrylate (PMMA) microparticles with diameter between 50 μm and 200 μm which dispersed in the water containing 0.3 wet% Pluronic F-127.

### Theoretical analysis for ABH microneedle acoustofluidic system

When the acoustic waves enter the fluid, the acoustic energy dissipates and attenuates in the fluid. The resulting second-order nonlinear effects are responsible for microparticle manipulation. One of these effects is acoustic radiation force (ARF), which is caused by scatting at the interface between two media with different acoustic properties. Thus, the microparticles dispersed in the fluid is affected by the ARF, which can be calculated as follows^56,57^

1 $${{\bf{F}}}_{{\bf{ARF}}}=\frac{-4\pi }{3}{{r}_{p}}^{3}\left[\frac{{f}_{1}{k}_{f}{p}_{1}\ast \nabla {p}_{1}}{2}-\frac{3{f}_{2}{\rho }_{f}{{\bf{v}}}_{{\bf{1}}}\ast \nabla {{\bf{v}}}_{{\bf{1}}}}{4}\right]$$

2 $${f}_{1}(\tilde{k})=1-\tilde{k},{\rm{with}}\,\tilde{k}=\frac{{k}_{p}}{{k}_{f}}$$

3 $${f}_{2}(\tilde{\rho })=\frac{2(\tilde{\rho }-1)}{2\tilde{\rho }+1},{\rm{with}}\,\tilde{\rho }=\frac{{\rho }_{p}}{{\rho }_{f}},$$

where *r*_p_ is the radius of microparticles, *k*_p_ and *k*_f_ is the compressibility of microparticle and fluid, respectively, *p*_1_ is the acoustic pressure, *ρ*_p_ and *ρ*_f_ is the density of microparticle and fluid, **v**_1_ is the velocity field of the wave.

The other effect is called acoustic streaming, which is due to the attenuation of an acoustic wave and its momentum transfer to the fluid. This effect acts on the microparticles via drag force, which can be calculated as follows^58,59^

4 $${{\bf{F}}}_{{\bf{drag}}}=6\pi \mu {r}_{p}{\bf{u}}$$

where *μ* is the dynamic viscosity of fluid, **u** is velocity of the surrounding flows relative to that the particle. Taking the above factors into consideration, we can design the acoustic particle manipulation system.

### Numerical simulation for ABH microneedle acoustofluidic system

In order to study the principle of the ABH microneedle acoustofluidic system in microparticle manipulation, we numerically simulated the acoustic pressure and acoustic streaming flows with the FEM software COMSOL Multiphysics in frequency domain, similar as a previously published method^60^. In order to simplify the calculation process, we used a two-dimensional model, which consisted of a plastic ABH microneedle, a container filled with water. And the profile curve of the ABH was designed as ${y}=\frac{5000}{{(500-5)}^{\frac{1}{2}}}{({x}-5)}^{\frac{1}{2}}$. The *x* is the radius of ABH, thus, the diameter in the tip is 10 μm, and 1000 μm in the top. A square area of 1 mm by 1 mm was designed under the tip of the needle for calculating the average of ARF and acoustic flow velocity. Furthermore, we demonstrated a computational meshing scheme that achieves advisable accuracy in suitable calculation time. For the part of the microneedle in the air, the maximum element size of Free Quad was set as 0.1 mm to ensure divide the needle into 10 parts. For the part of the microneedle in the water where diameter from 1000 μm to 100 μm, the maximum element of Free Triangular was set as 10 μm. Besides, at the tip of the microneedle, the maximum element was set as 1 μm to ensure that the tip was divided to 10 parts. And for the water domain, the maximum element was set as 100 μm to ensure the size was less than 0.1 of wavelength at the working frequency. Afterwards, we used this model to perform simulation calculation. The whole process can be divided into three steps.

First, the Solid Mechanics and Pressure Acoustics module in the frequency domain was employed to solve the acoustic field produced by piezoelectric transducer and propagating along the microneedle to the water. The governing equation used in the module is as follows

5 $$-{\rho }_{s}{\omega }^{2}{\bf{u}}=\nabla \cdot {\bf{S}}+{{\bf{F}}}_{{\bf{v}}}{e}^{i\phi }$$

6 $${\nabla }^{2}{p}_{1}+\frac{{w}^{2}}{{{c}_{f}}^{2}}{p}_{1}=0$$

where *ρ*_s_ is the density of solid microneedle. *ω* is the angular frequency, **u** is the displacement vector, **S** is the second Piola-Kirchhoff stress, **F**_**v**_ is the volume force vector, i is the imaginary unit, *ϕ* is the phase angle. *c*_f_ is the wave speed in the fluid.

After solving the acoustic field, the body force calculated from the Thermoviscous Acoustics module was substituted to the Laminar Flow module to calculate acoustic streaming profile. The governing equations used in the module are the continuity equation based on mass conservation and the Navier-Stokes equation based on momentum conservation, as shown below

7 $${\rho }_{f}\nabla \cdot {{\bf{v}}}_{{\bf{2}}}=0$$

8 $${\rho }_{f}({{\bf{v}}}_{{\bf{2}}}\cdot \nabla ){{\bf{v}}}_{{\bf{2}}}=-\nabla {p}_{2}+\mu {\nabla }^{2}{{\bf{v}}}_{{\bf{2}}}+\left({\mu }_{b}+\frac{1}{3}\mu \right)\nabla (\nabla \cdot {{\bf{v}}}_{{\bf{2}}})+{\bf{F}}$$

9 $${\bf{F}}=-{\rho }_{f}\left\langle ({{\bf{v}}}_{{\bf{1}}}\cdot \nabla ){{\bf{v}}}_{{\bf{1}}}+{{\bf{v}}}_{{\bf{1}}}\left(\nabla \cdot {{\bf{v}}}_{{\bf{1}}}\right)\right\rangle$$

10 $${{\bf{v}}}_{{\bf{1}}}=\frac{\nabla {p}_{1}}{iw{\rho }_{f}}$$

where *ρ*_f_ is the density of fluid, **v**_**2**_ is the acoustic streaming velocity in the fluid, *p*_2_ is the pressure in the fluid, *μ*_b_ is the bulk viscosity, **F** is the body force relative to **v**_**1**_ of time-harmonic acoustic particle velocity.

## Supplementary information

supplemental material
