# Supplementary material for: Acoustic black hole effect enhanced micro-manipulator
Source: Microsyst Nanoeng. 2024 Oct 12;10:144. doi: 10.1038/s41378-024-00789-z (PMC11470035; doi:10.1038/s41378-024-00789-z)
Supplement: Supplementary file 1 — supplemental material [file 41378_2024_789_MOESM1_ESM.docx]

Supplementary Materials for

**Acoustic black hole effect enhanced micro-manipulator**

Qiu Yin^1, 2^, Haoyong Song^3^, Zhaolong Wang^4^, Zhichao Ma^2*^, Wenming Zhang^1*^

1. State Key Laboratory of Mechanical System and Vibration, Shanghai Jiao Tong University, Shanghai 200240, China

2. Institute of Medical Robotics, School of Biomedical Engineering, Shanghai Jiao Tong University, Shanghai 200240, China

3. College of Mechanical and Vehicle Engineering, Hunan University, Changsha 410082, China

4. School of Energy Science and Engineering, Harbin Institute of Technology, Harbin 150001, P. R. China

*Correspondence: wenmingz@sjtu.edu.cn (W.M. Zhang); zhichaoma@sjtu.edu.cn （Z.C. Ma）

**This PDF file includes:**

Fig. S1 to S2


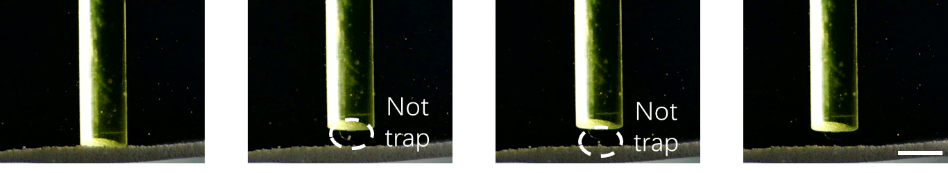


Fig.S1 The experiment shows that cylindrical microneedle can not trap particle. Scale bar: 1000 μm.

Fig.S2 The relationship between the average vibration velocity and operating frequency of the ABH microneedle at 1Vpp.
